# Supplementary material for: Short Assessment for People with Human Immunodeficiency Virus (HIV) Aged 50 Years or Older: Essential Tests from Comprehensive Geriatric Assessment
Source: Viruses. 2025 Jun 24;17(7):887. doi: 10.3390/v17070887 (PMC12300530; doi:10.3390/v17070887)
Supplement: Supplementary file 1 [file viruses-17-00887-s001.zip › viruses-3645305-supplementary.pdf]

Supplementary file

Table S1. Number of abnormal test results from the short CGA according to HIV infection status, *N (%)*.

	**Overall (N=345)**	**Control (N=55)**	**PWH (N=290)**
0	113 (32.75)	22 (40.00)	91 (31.38)
1	133 (38.55)	17 (30.91)	116 (40.00)
≥2	99 (28.70)	16 (29.09)	83 (28.62)

CGA, comprehensive geriatric assessment; HIV, human immunodeficiency virus; PWH, people with HIV.

Table S2. Baseline characteristics of study patients according to HIV infection status and number of abnormal test results from the short CGA.

	Control						PWH					
	**No abnormal results (N=22)**	**N**	**1 abnormal result (N=17)**	**N**	**≥2 abnormal results (N=16)**	**N**	**No abnormal results (N=91)**	**N**	**1 abnormal result (N=116)**	**N**	**≥2 abnormal results (N=83)**	**N**
**Sociodemographic characteristics**												
Age (years), *mean (SD)*	63.41 (5.85)	22	63.41 (7.60)	17	70.44 (8.82)	16	61.32 (6.71)	90	64.60 (9.08)	116	67.17 (10.22)	83
Males, *n (%)*	18 (81.82)	22	9 (52.94)	17	7 (43.75)	16	78 (85.71)	91	89 (76.72)	116	57 (68.67)	83
Education level, *n (%)*		22		17		16		91		116		83
Uneducated	0 (0.00)		2 (11.76)		4 (25.00)		3 (3.30)		8 (6.90)		17 (20.48)	
Primary/secondary education	3 (13.64)		9 (52.94)		11 (68.75)		43 (47.25)		66 (56.90)		43 (51.81)	
Higher education	19 (86.36)		6 (35.29)		1 (6.25)		45 (49.45)		42 (36.21)		23 (27.71)	
Employment status, *n (%)*		22		17		16		91		116		83
Unemployed	0 (0.00)		4 (23.53)		4 (25.00)		1 (1.10)		19 (16.38)		17 (20.48)	
Active	13 (59.09)		8 (47.06)		5 (31.25)		47 (51.65)		61 (52.59)		37 (44.58)	
Retired or pensioner	9 (40.91)		1 (5.88)		0 (0.00)		40 (43.96)		22 (18.97)		25 (30.12)	
Other	0 (0.00)		4 (23.53)		7 (43.75)		3 (3.30)		14 (12.07)		4 (4.82)	
Cohabitation status, *n (%)*		22		17		16		91		116		83
Living with partner	14 (63.64)		12 (70.59)		8 (50.00)		47 (51.65)		52 (44.83)		30 (36.14)	
Living with a relative	5 (22.73)		4 (23.53)		3 (18.75)		14 (15.38)		18 (15.52)		12 (14.46)	
Living with friends	0 (0.00)		0 (0.00)		1 (6.25)		6 (6.59)		11 (9.48)		8 (9.64)	
Living alone	3 (13.64)		1 (5.88)		4 (25.00)		24 (26.37)		35 (30.17)		33 (39.76)	
**Clinical characteristics**												
Body mass index (kg/m2), *median (IQR)*	27.29 (23.51, 28.57)	22	27.23 (24.22, 27.77)	17	29.43 (25.58, 31.16)	16	25.68 (23.75, 27.79)	91	24.77 (23.01, 28.22)	113	24.89 (22.28, 28.21)	83
Blood pressure (mmHg), *median (IQR)*		21		17		16		91		102		80
Systolic	141.00 (128.00, 150.00)		126.00 (115.00, 153.00)		153.00 (139.00, 176.00)		136.00 (121.00, 151.50)		135.00 (123.00, 148.00)		129.50 (116.00, 143.00)	
Diastolic	82.00 (78.00, 92.00)		80.00 (72.00, 86.00)		93.50 (91.00, 97.25)		82.00 (77.00, 89.00)		80.00 (74.00, 85.00)		77.00 (72.00, 84.00)	

Number of specialists consulted, *n (%)*		13		5		11		62		59		57
0	2 (15.38)		0 (0.00)		0 (0.00)		13 (20.97)		23 (38.98)		14 (24.56)	
1	9 (69.23)		4 (80.00)		10 (90.91)		29 (46.77)		24 (40.68)		27 (47.37)	
≥2	2 (15.38)		1 (20.00)		1 (9.09)		20 (32.26)		12 (20.34)		16 (28.07)	
Number of admissions during the last year, *n (%)*		22		17		15		91		116		83
0	20 (90.91)		17 (100.00)		15 (100.00)		82 (90.11)		111 (95.69)		71 (85.54)	
≥1	2 (9.09)		0 (0.00)		0 (0.00)		9 (9.89)		5 (4.31)		12 (14.46)	
Number of falls during the last year, *n (%)*		22		17		15		90		116		83
0	21 (95.45)		16 (94.12)		15 (100.00)		81 (90.00)		102 (87.93)		64 (77.11)	
≥1	1 (4.55)		1 (5.88)		0 (0.00)		9 (10.00)		14 (12.07)		19 (22.89)	
Early menopause (<45 years old), *n (%)*	0 (0.00)	4	6 (75.00)	8	9 (100.00)	9	0 (0.00)	13	9 (33.33)	27	10 (38.46)	26
Smoking habit, *n (%)*		22		17		16		91		116		80
Non-smoker	15 (68.18)		6 (35.29)		8 (50.00)		31 (34.07)		32 (27.59)		29 (36.25)	
Current smoker	2 (9.09)		6 (35.29)		6 (37.50)		31 (34.07)		49 (42.24)		28 (35.00)	
Former smoker (>6 months)	5 (22.73)		5 (29.41)		2 (12.50)		29 (31.87)		35 (30.17)		23 (28.75)	
Alcohol consumption (>3 units/day), *n (%)*	4 (18.18)	22	0 (0.00)	17	2 (12.50)	16	7 (7.78)	90	14 (12.07)	116	10 (12.05)	83
Number of comorbidities, *median (IQR)*	3.00 (1.00, 3.00)	22	1.00 (1.00, 2.00)	17	2.00 (1.50, 3.50)	16	3.00 (2.00, 5.50)	91	3.00 (2.00, 4.25)	116	3.00 (2.00, 5.00)	83
Fecal incontinence, *n (%)*	0 (0.00)	22	1 (5.88)	17	1 (6.25)	16	2 (2.20)	91	0 (0.00)	115	7 (8.54)	82
Constipation/defecation urgency, *n (%)*	0 (0.00)	22	1 (5.88)	17	1 (6.25)	16	3 (3.33)	90	7 (6.03)	116	10 (12.35)	81

CGA, comprehensive geriatric assessment; HIV, human immunodeficiency virus; PWH, people with HIV.

Table S3. Abnormal test results from the original CGA according to HIV infection status and number of abnormal test results from the short CGA, *N (%)*.

	**Control**						**HIV**					
	**No abnormal results (N=22)**	**N**	**1 abnormal test result (N=17)**	**N**	**≥2 abnormal test results (N=16)**	**N**	**No abnormal results (N=91)**	**N**	**1 abnormal test result (N=116)**	**N**	**≥2 abnormal test results (N=83)**	**N**
Barthel Index	0 (0.00)	22	0 (0.00)	17	0 (0.00)	16	0 (0.00)	91	0 (0.00)	113	1 (1.22)	82
Lawton Score	0 (0.00)	22	15 (88.24)	17	16 (100.00)	16	0 (0.00)	91	73 (62.93)	116	63 (75.90)	83
Hearing-Dependent Daily Activities scale	10 (45.45)	22	4 (23.53)	17	5 (31.25)	16	40 (44.44)	91	60 (51.72)	116	47 (58.75)	80
Lagro-Janssen index	-	0	0 (0.00)	15	0 (0.00)	15	2 (66.67)	3	3 (4.11)	73	7 (11.86)	59
Pittsburgh Sleep Quality Index	0 (0.00)	22	0 (0.00)	17	0 (0.00)	16	0 (0.00)	91	4 (3.45)	116	13 (15.66)	83
Fried criteria	0 (0.00)	22	1 (6.25)	16	4 (25.00)	16	4 (4.40)	91	10 (10.99)	91	17 (26.98)	63
Short Physical Performance Battery	0 (0.00)	22	1 (5.88)	17	15 (93.75)	16	0 (0.00)	91	12 (10.34)	116	49 (59.04)	83
Mini Nutritional Assessment-Short Form	1 (5.26)	19	0 (0.00)	15	0 (0.00)	15	18 (22.78)	79	29 (26.61)	109	29 (36.25)	80
Barber questionnaire	0 (0.00)	22	0 (0.00)	17	4 (25.00)	16	0 (0.00)	91	7 (6.03)	116	33 (39.76)	83
Functional Ambulation Categories	1 (4.55)	22	0 (0.00)	2	0 (0.00)	1	0 (0.00)	90	0 (0.00)	43	6 (20.00)	30
Geriatric Depression Scale	1 (4.76)	21	0 (0.00)	2	1 (100.00)	1	12 (13.48)	89	18 (41.86)	43	22 (73.33)	30
Cognitive Complaints questionnaire	0 (0.00)	22	1 (5.88)	17	2 (12.50)	16	0 (0.00)	91	20 (17.24)	116	46 (55.42)	83
NEUrocognitive Screen	3 (14.29)	21	1 (50.00)	2	1 (50.00)	2	18 (19.78)	91	10 (24.39)	41	12 (48.00)	25
Cognitive Reserve Questionnaire		20		2		1		88		43		29
Lower range	0 (0.00)		0 (0.00)		0 (0.00)		9 (10.23)		5 (11.63)		6 (20.69)	
Medium–low range	1 (5.00)		1 (50.00)		1 (100.00)		11 (12.50)		8 (18.60)		5 (17.24)	
Medium–high range	5 (25.00)		0 (0.00)		0 (0.00)		29 (32.95)		15 (34.88)		10 (34.48)	
Upper range	14 (70.00)		1 (50.00)		0 (0.00)		39 (44.32)		15 (34.88)		8 (27.59)	

CGA, comprehensive geriatric assessment; HIV, human immunodeficiency virus; PWH, people with HIV.

Table S4: Clinical Frailty Score (CFS) in function of the value of the short assessment

	Overall						Control						VIH					
	**None altered** N = 113	**N**	**1 altered** (N = 133)	**N**	**2+ altered** (N = 99)	**N**	**None altered** (N = 22)	**N**	**1 altered** (N = 17)	**N**	**2+ altered** (N = 16)	**N**	**None altered** (N = 91)	**N**	**1 altered** (N = 116)	**N**	**2+ altered** (N = 83)	**N**
Clinical Frailty Score (CFS)		113		45		31		22		2		1		91		43		30
Fit	45 (39.82%)		10 (22.22%)		1 (3.23%)		10 (45.45%)		1 (50.00%)		0 (0.00%)		35 (38.46%)		9 (20.93%)		1 (3.33%)	
Good Health	46 (40.71%)		14 (31.11%)		2 (6.45%)		9 (40.91%)		0 (0.00%)		0 (0.00%)		37 (40.66%)		14 (32.56%)		2 (6.67%)	
Proper handling	22 (19.47%)		13 (28.89%)		9 (29.03%)		3 (13.64%)		0 (0.00%)		1 (100.00%)		19 (20.88%)		13 (30.23%)		8 (26.67%)	
Vulnerable	0 (0.00%)		7 (15.56%)		13 (41.94%)		0 (0.00%)		1 (50.00%)		0 (0.00%)		0 (0.00%)		6 (13.95%)		13 (43.33%)	
Slightly fragile	0 (0.00%)		0 (0.00%)		0 (0.00%)		0 (0.00%)		0 (0.00%)		0 (0.00%)		0 (0.00%)		0 (0.00%)		0 (0.00%)	
Moderately fragile	0 (0.00%)		1 (2.22%)		6 (19.35%)		0 (0.00%)		0 (0.00%)		0 (0.00%)		0 (0.00%)		1 (2.33%)		6 (20.00%)	
Severely fragile	0 (0.00%)		0 (0.00%)		0 (0.00%)		0 (0.00%)		0 (0.00%)		0 (0.00%)		0 (0.00%)		0 (0.00%)		0 (0.00%)	
Very severely fragile	0 (0.00%)		0 (0.00%)		0 (0.00%)		0 (0.00%)		0 (0.00%)		0 (0.00%)		0 (0.00%)		0 (0.00%)		0 (0.00%)	
